# Preoperative Hemodynamics and Brain Injury in Transposition of the Great Arteries

**DOI:** 10.1016/j.jacadv.2026.102592

**Published:** 2026-03-25

**Authors:** Devin Chetan, Thiviya Selvanathan, Fu-Tsuen Lee, Hanan Smaili, Min Bao, Jessie Mei Lim, Davide Marini, Amandeep Saini, Shabnam Peyvandi, Patrick McQuillen, Helen M. Branson, Vann Chau, Steven P. Miller, Mike Seed

**Affiliations:** a Division of Cardiology, The Labatt Family Heart Centre, The Hospital for Sick Children, Toronto, Ontario, Canada; b Department of Pediatrics, University of Toronto, Toronto, Ontario, Canada; c Division of Pediatric Cardiology, Lillehei Heart Institute, Masonic Children’s Hospital, Minneapolis, Minnesota, USA; d Department of Pediatrics, University of Minnesota, Minneapolis, Minnesota, USA; e Department of Pediatrics, University of British Columbia, Vancouver, British Columbia, Canada; f BC Children’s Hospital Research Institute, Vancouver, British Columbia, Canada; g Division of Neurology, The Hospital for Sick Children, Toronto, Ontario, Canada; h Department of Physiology, Temerty Faculty of Medicine, University of Toronto, Toronto, Ontario, Canada; i Division of Pediatric Cardiology, Department of Pediatrics and Epidemiology & Biostatistics, Benioff Children's Hospital, University of California-San Francisco, San Francisco, California, USA; j Division of Critical Care, Department of Pediatrics, Benioff Children's Hospital, University of California-San Francisco, San Francisco, California, USA; k Department of Diagnostic Imaging and Interventional Radiology, The Hospital for Sick Children, Toronto, Ontario, Canada; l Department of Medical Imaging, University of Toronto, Toronto, Ontario, Canada

**Keywords:** cardiac MRI, cerebrovascular circulation, heart-brain, hemodynamics, transitional circulation, transposition of the great arteries

## Abstract

**Background:**

Despite improvements in transposition of the great arteries (TGA) outcomes, perinatal brain injury remains common.

**Objectives:**

The purpose of this study was to document patterns of brain injury and to explore hemodynamic mechanisms of brain injury in a subset of patients using cardiovascular magnetic resonance (CMR).

**Methods:**

Preoperative brain magnetic resonance imaging and CMR were performed between 2014 and 2023. Brain injury was classified as stroke, white matter injury, or hypoxic ischemic injury. Spearman’s correlation was used to assess relationships between hemodynamic parameters.

**Results:**

Neuroimaging was obtained in 225/285 (79%) neonates with TGA, of whom 45/225 (20%) underwent CMR. Brain injury included arterial ischemic stroke (22/225; 9.8%), moderate-to-severe white matter injury (19/225; 8.4%), and hypoxic ischemic injury (6/225; 2.7%). Higher ratio of pulmonary to systemic blood flow (QpQs) was associated with lower superior vena cava flow and cerebral oxygen delivery but not cerebral blood flow. Higher QpQs was also associated with a higher ratio of cerebral blood flow to systemic blood flow. Hypoxic ischemic brain injuries occurred early due to a restrictive atrial septum or later, following pulmonary overcirculation.

**Conclusions:**

Although no clear association was found with brain injury, this study provides evidence that high QpQs is related to lower superior vena cava flow and cerebral oxygen delivery and may predispose infants with TGA to white matter injury and hypoxic ischemic injury. These findings suggest that to preserve cerebral blood flow, preoperative TGA patients who remain hypoxemic after balloon atrial septostomy may be better served by earlier arterial switch operation rather than attempting to increase patent ductus arteriosus size with prostaglandins. Our findings suggest hypoxic ischemic injury secondary to pulmonary overcirculation is also an important form of injury and may be preventable through judicious use of prostaglandin and timely surgical repair.

Recent reports reveal excellent outcomes for children with d-transposition of the great arteries (TGA), having mortality of 5% or less at 10 years following repair.^1^^,^^2^ Nevertheless, patients with TGA remain at risk for neurodevelopmental disability.^3^^,^^4^ Immaturity of the neonatal brain resulting from abnormal fetal circulatory physiology is thought to predispose newborns with TGA to patterns of brain injury that are more typical of preterm infants, including white matter injury; moderate-to-severe white matter injury has been linked to adverse neurodevelopmental outcomes.^5^, ^6^, ^7^, ^8^ Operative factors may be less important than innate patient factors and preoperative management as contributors to brain injury, highlighting the importance of the transitional circulation.^9^^,^^10^ Severe brain injury has been described in TGA patients presenting with severe cyanosis and circulatory collapse early after birth.^11^, ^12^, ^13^ This complication results from the restriction of atrial-level shunting of oxygenated blood from the left atrium into the systemic circulation due to perinatal closure or pre-existing restriction of the foramen ovale.

Prenatal diagnosis of TGA with planned delivery at centers with expertise in undertaking urgent balloon atrial septostomy (BAS) has significantly reduced the risk of brain injury from a restrictive atrial septum.^14^ BAS has itself been associated with arterial ischemic stroke, likely due to thrombus caused by the procedure into the systemic arterial circulation or the embolization of air introduced into the systemic venous system.^15^ Studies have not shown the same risk of stroke with BAS likely attributable to meticulous attention to technique when accessing the circulation.^16^^,^^17^ Routine administration of prostaglandin in the delivery room has also helped to prevent severe cyanosis by allowing a left-to-right shunt from the systemic to the pulmonary circulation, thereby increasing pulmonary blood flow, left atrial return and left-to-right shunting at atrial level. However, with the expected fall in pulmonary vascular resistance following birth, the progression of predominantly left-to-right shunts at the ductus arteriosus and foramen ovale following atrial septostomy and prostaglandin infusion may result in excessive pulmonary blood flow and a risk of systemic hypoperfusion, as is recognized in the setting of other forms of congenital heart disease such as hypoplastic left heart syndrome.^18^ The impact of excessive pulmonary blood flow on systemic perfusion may overcome cerebral autoregulation and contribute to the risk of brain injury.^10^

The prevalence of perinatal brain injury in patients undergoing neonatal cardiac repairs has resulted in the application of routine neuroimaging in this population.^19^ With improved access to magnetic resonance imaging (MRI) at many pediatric centers undertaking congenital heart surgery, this modality has largely superseded the use of cranial ultrasound and has been shown to detect common forms of brain injury with greater accuracy.^20^ Similarly, the utility of cine phase-contrast MRI for the noninvasive quantification of vessel flow has been recognized as a useful tool for assessing the hemodynamics of congenital heart disease. We report a combination of findings from the clinical implementation of routine neonatal brain MRI in patients with TGA at our center, with a prospective research study in which preoperative hemodynamics were also studied in a sample of these patients using cardiovascular magnetic resonance (CMR).

## Methods

### Study design

The “Cardiac Neurodevelopment Study” was a single-center prospective observational study examining the relationship between cardiovascular physiology and brain development in patients with TGA and single ventricle physiology enrolled at The Hospital for Sick Children from 2016 to 2019. TGA patients consecutively enrolled in this study underwent preoperative brain and cardiac MRI, the results of which are described below. The neuroimaging findings reported here are also nested in a larger retrospective cohort of patients with TGA cared for at our center between 2014 and 2023, the majority of whom underwent preoperative brain MRI as part of a routine clinical initiative. The objectives of the present study were to: 1) describe brain MRI findings in a large cohort of patients with TGA; 2) examine detailed cardio-cerebral hemodynamics in a smaller subset with CMR; and 3) explore important associations between hemodynamic variables and their possible relationship to brain injury.

### Patient population

The Hospital for Sick Children is the provincial center for TGA surgery in Ontario and undertakes ∼30 arterial switch operations performed in our province each year. Approximately half of the TGA patients born in Ontario are currently diagnosed prenatally and are typically delivered at term at a tertiary obstetric center near the pediatric hospital and transferred to our cardiac critical care unit soon after birth. An infusion of prostaglandin E1 (PGE) is usually initiated in the delivery room. Transthoracic echocardiography is performed upon arrival in the intensive care unit. Urgent BAS is undertaken by a pediatric interventional cardiologist in the intensive care unit or nearby cardiac catheterization laboratory when the preductal arterial oxygen saturations are below 75% or if the foramen ovale is felt to be restrictive. Following BAS, prostaglandins are discontinued except with arterial desaturation below 75%. The arterial switch operation is usually performed during the first week of life (Table 1, Supplemental Tables 1 and 2). Surgery may be delayed by preterm birth, postnatal diagnosis, associated lesions, or preoperative complications. In the operating room, all patients undergo median sternotomy, cardiopulmonary bypass, and aortic cross-clamp with mild hypothermia. Circulatory arrest is used infrequently. Genetic testing is performed in the setting of multiple congenital malformations or dysmorphic features. Using a combination of chart review and prospective data collection, we obtained information regarding the clinical history of the patients in our cohort, including their demographic data, delivery room and critical care course, as well as hemodynamic status such as their vital signs and any complications that might be associated with circulatory instability.

**Table 1:** Cohort Demographics

	Cohort (n = 285)	No CMR (n = 240)	CMR (n = 45)	*P* Value
Gestational age, wk	39.0 (38.1, 39.9)	39.0 (38.1, 39.8)	39.1 (38.0, 40.0)	0.42
Male:female	195/285 (68%):90/285 (32%)	165/240 (69%):75/240 (31%)	30/45 (67%):15/45 (33%)	0.92
Birth weight (kg)	3.25 (2.95, 3.57)	3.25 (2.94, 3.57)	3.26 (3.00, 3.52)	0.71
Antenatal diagnosis	165/285 (58%)	141/240 (59%)	24/45 (53%)	0.61
Prematurity	23/285 (8%)	22/240 (9%)	1/45 (2%)	0.09
Very preterm (28-31^6^)	4/285 (1%)	4/240 (2%)	-	
Moderate preterm (32-33^6^)	2/285 (1%)	2/240 (1%)	-	
Late preterm (34-36^6^)	17/285 (6%)	16/240 (7%)	1/45 (2%)	
Septostomy	198/285 (69%)	158/240 (66%)	40/45 (89%)	0.004
Age at septostomy (days)	0 (0, 1)	0 (0, 1)	1 (0, 1)	0.81
Diagnosis^a^				0.66
TGA/IVS	157/285 (55%)	130/240 (54%)	27/45 (60%)	
TGA/VSD	100/285 (35%)	83/240 (35%)	17/45 (38%)	
Double outlet right ventricle + TGA	8/285 (3%)	8/240 (3%)	0/45 (0%)	
Taussig-Bing	17/285 (6%)	16/240 (7%)	1/45 (2%)	
Other	3/285 (1%)	3/240 (1%)	0/45 (0%)	
Brain injury				0.005
Arterial ischemic stroke	22/225 (9.8%)	16/180 (8.9%)	6/45 (13.3%)	
White matter injury	52/225 (23.1%)	47/180 (26.1%)	5/45 (11.1%)	
Mild	33/225 (14.7%)	33/180 (18.3%)	0/45 (0%)	
Moderate	16/225 (7.1%)	11/180 (6.1%)	5/45 (11.1%)	
Severe	3/225 (1.3%)	3/180 (1.7%)	0/45 (0%)	
Hypoxic ischemic injury	6/225 (2.7%)	5/180 (2.8%)	1/45 (2.2%)	
Basal ganglia + watershed	3/225 (1.3%)	3/180 (1.7%)	0/45 (0%)	
Watershed	3/225 (1.3%)	2/180 (1.1%)	1/45 (2.2%)	
Grade 3 intraventricular hemorrhage/PVHI	1/225 (0.4%)	1/180 (0.6%)	0/45 (0%)	
Age at surgery (d)	7 (5, 14)	7 (5, 15)	7 (6, 9)	0.61
Weight at surgery (kg)	3.41 (3.02, 3.90)	3.40 (3.02, 3.98)	3.42 (3.05, 3.64)	0.45
Body surface area at surgery (m^2^)	0.23 (0.21, 0.26)	0.23 (0.21, 0.26)	0.24 (0.21, 0.26)	0.82
Cardiopulmonary bypass time (min)	147 (123, 185)	147 (123, 185)	146 (120, 170)	0.65
Aortic cross clamp time (min)	93.5 (78, 122.8)	92 (77, 121)	98 (83, 129)	0.43
Type of surgery^a^				0.57
Arterial switch operation (+/− VSD, arch)	263/285 (92%)	218/240 (90.8%)	45/45 (100%)	
Rastelli	12/285 (4%)	12/240 (5.0%)	0/45 (0%)	
Nikaidoh	4/285 (1%)	4/240 (1.7%)	0/45 (0%)	
Other	2/285 (1%)	2/240 (0.8%)	0/45 (0%)	
Died before surgery	4/285 (1%)	4/240 (1.7%)	0/45 (0%)	
30-day mortality	8/285 (3%)	6/240 (3%)	2/45 (4%)	0.53

IVS = intact ventricular septum; PVHI = periventricular hemorrhagic infarction; TGA = transposition of the great arteries; VSD = ventricular septal defect.

a Detailed diagnoses and surgical details in Supplemental Tables 1 and 2.

### Preoperative brain MRI

The preoperative brain MRI protocol was the same for patients undergoing imaging as part of our clinical protocol or as part of the study and was usually performed in the first few days of life on a 1.5-T clinical MRI system (Siemens AvantoFit) with a dedicated neonatal head coil. Patients were scanned without sedation during natural sleep and monitored throughout by a physician and nurse with experience caring for newborns with critical congenital heart disease using a temperature probe and pulse oximetry. The sequences acquired included high resolution three-dimensional T1-weighted gradient echo obtained in a sagittal plane and reconstructed in axial and coronal reformats, axial diffusion-weighted imaging, T2-weighted spin echo, susceptibility-weighted imaging, and MR venography. The details of the sequences are provided in the Supplemental Table 3.

**Figure 1:**
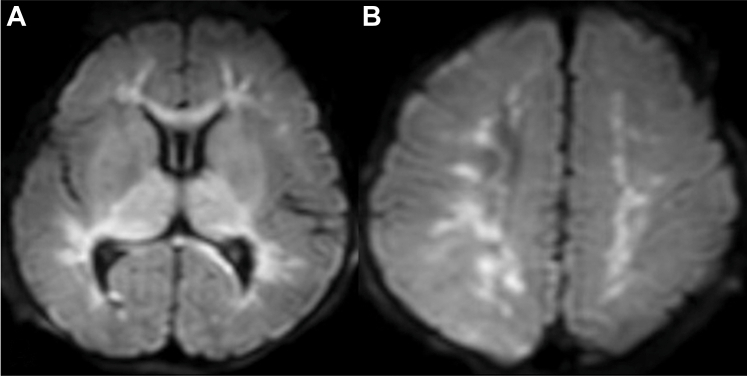
**Preoperative Brain MRI in a 2-Day Old Infant** (A and B) Axial diffusion weighted imaging showing injury to the subcortical, periventricular, and deep white matter, internal capsule, thalami and basal ganglia bilaterally consistent with hypoxic-ischemic brain injury. MRI = magnetic resonance imaging.

The brain MRI imaging was reported clinically by expert pediatric neuroradiologists and further reviewed by a neonatal neurologist (T.S.). For the purposes of this analysis, parenchymal brain injuries were classified into one of 4 patterns. Arterial ischemic strokes were defined as discrete lesions exhibiting restricted diffusion and limited to an expected vascular territory, while white matter injuries were defined as T1 hyperintense punctate lesions located in the periventricular white matter. Severity was scored based on the number and size of white matter lesions as described previously.^21^ Larger, less well-defined areas of restricted diffusion affecting both hemispheres and centered on watershed areas between the expected territories of the major arteries and/or deep gray structures were defined as hypoxic ischemic injury (Figure 1). Significant intraventricular hemorrhage was graded according to the Papile classification.^22^ Total brain volumes were calculated using three-dimensional steady-state free precession or T1-weighted gradient echo acquisition images and brain segmentation using a commercial software package (Mimics, Materialize) as previously described.^23^ Total brain volumes were converted to estimated brain weights using a published conversion factor based on estimated brain density.^24^ We converted brain weights to postmenstrual age–appropriate *z*-scores according to a previously published large autopsy data set.^25^

### Preoperative cardiac MRI

CMR was undertaken in the study patients with a phased array surface coil with electrocardiographic triggering. High resolution cine phase-contrast measurements were acquired during free breathing with multiple repetitions in the ascending aorta, superior vena cava (SVC), main pulmonary artery, right and left pulmonary arteries, ductus arteriosus, and descending aorta. The flow measurements were processed using commercial segmentation software (QFlow, Medis). Flows were indexed to body surface area with flows expressed in L/min/m^2^.

The cardiac MRI findings were also reviewed by an experienced cardiac radiologist (M.S.). Systemic blood flow was calculated from a combination of systemic venous return from the upper body (SVC flow) and arterial supply to the lower body (descending aortic flow). Pulmonary blood flow was calculated as the sum of right and left pulmonary artery flows. SVC flow is known to account primarily for cerebral blood flow with only small contributions from the innominate and subclavian veins.^26^ We measured cerebral blood flow by using a slice prescription perpendicular to the right and left internal carotid arteries and basilar artery at the level of the clivus.

Measurements of the ductus arteriosus were taken if an angiogram was performed at the time of cardiac MRI or if scout images adequately visualized the ductus arteriosus. If a measurement of the ductus arteriosus was not available from the CMR images, a measurement from the closest echocardiogram was taken. Oxygen saturations were preductal and preferentially obtained during CMR where available or taken from retrospective review of the chart at the time closest to combined brain and cardiac MRI.

### Statistical analysis

Continuous variables were presented as median values with IQRs, while categorical variables were expressed as frequencies (percentages). Between-group comparisons of continuous variables were performed using the Mann-Whitney *U* test, and comparisons of categorical variables were conducted using Fisher exact test. Associations between hemodynamic parameters were evaluated using Spearman’s rank correlation coefficient to explore potential interrelationships among hemodynamic measures and their association with indicators of brain injury. Statistical analyses were conducted separately for patients with TGA/intact ventricular septum (IVS) and TGA/ventricular septal defect (VSD). Statistical analyses were conducted using GraphPad Prism version 10.3.0 (GraphPad Software Inc) and R version 4.5.1 (R Foundation for Statistical Computing). A 2-tailed *P* value of <0.05 was considered to indicate statistical significance.

## Results

### Cohort demographics

Over a 10-year period between 2014 and 2023, a total of 285 patients with TGA were treated at the Hospital for Sick Children, of which 225/285 (79%) underwent preoperative brain MRI and 45/225 (20%) underwent cardiac MRI. There were 4/285 (1%) patients who died prior to surgery and an additional 4/285 (1%) postoperative deaths prior to 30 days of age. Cardiac repairs consisted of an arterial switch operation (n = 263/285, 92%) ± VSD closure (n = 77/285, 27%) ± arch reconstruction (n = 23/285, 8%). There were 17/285 (6%) patients with pulmonary stenosis precluding an arterial switch operation. An antenatal diagnosis was made in 165/285 patients (58%). Prostaglandins were administered in almost all patients, while 198/285 (69%) underwent BAS. Cohort demographics are outlined in Table 1.

### Preoperative brain injury

Among the 225 patients with preoperative brain MRIs, brain injury included arterial ischemic stroke in 22/225 (9.8%), mild/moderate/severe white matter injury in 33/225 (14.7%)/16/225 (7.1%)/3/225 (1.3%), hypoxic ischemic injury in basal ganglia and watershed regions in 3/225 (1.3%), watershed only in 3/225 (1.3%), grade 3 intraventricular hemorrhage or periventricular hemorrhagic infarction in 1/225 (0.4%) patients. Among the subset undergoing cardiac MR, 11/45 (24.4%) had brain injury 6/45 (13.3%) with arterial ischemic stroke, 5/45 (11.1%) with moderate white matter injury (one patient had stroke and white matter injury), and 1/45 (2.2%) with watershed only hypoxic ischemic injury.

Among the 45 patients undergoing CMR, there were no significant differences in preoperative demographics, prostaglandin use, septostomy, brain weights or volumes, or age at preoperative MRI between patients with and without brain injury (Table 2). Two of these patients had early profound hemodynamic instability and required venoarterial extracorporeal membrane oxygenation. The first patient (TGA/IVS, antenatal diagnosis) was flat at birth and profoundly hypoxic upon transfer from the tertiary obstetric facility. There was minimal improvement despite extensive resuscitation efforts including a failed attempt at BAS. The infant was subsequently cannulated onto extracorporeal membrane oxygenation for 3 days. This patient was found to have watershed hypoxic ischemic injury on preoperative MRI. The second patient (TGA/IVS, postnatal diagnosis) presented in shock shortly after birth. The patient was cannulated following resuscitative efforts and underwent BAS a few hours after being cannulated. The patient was decannulated from extracorporeal membrane oxygenation after about 10 hours having been noted on cranial ultrasound to have suffered a watershed intraparenchymal hemorrhage. Subsequent brain MRI showed a right middle cerebral artery territory infarction. There were no significant differences in operative variables including age, weight, body surface area, cardiopulmonary bypass time, and aortic cross-clamp time between patients with and without brain injury (Table 2). Patients with brain injury had similar duration of mechanical ventilation after surgery (*P* = 0.19), longer intensive care unit stay (*P* = 0.04), and trend toward longer hospital stay (*P* = 0.07). Patients with septostomy did not have a higher incidence of arterial ischemic stroke (septostomy: 17 of 194 (9%) vs no septostomy: 5 of 87 (6%); *P* = 0.41).

**Table 2:** Profile of Patients Undergoing CMR Stratified by Presence of Brain Injury

	Cohort (n = 45)	No Brain Injury (n = 34)	Brain Injury (n = 11)	*P* Value
Preoperative variables				
Birth gestational age (wk)	39.1 (38.0, 40.0)	39.0 (38.0, 40.3)	39.3 (38.7, 39.4)	0.71
Male	30/45 (67%)	21/34 (62%)	9/11 (82%)	0.29
Apgar score (1 min)	8 (7, 8)	8 (7, 9)	8 (7, 8)	0.45
Apgar score (5 min)	8 (8, 9)	8 (8, 9)	9 (8, 9)	0.34
Birth weight (kg)	3.26 (3.00, 3.52)	3.22 (2.93, 3.50)	3.52 (3.16, 3.68)	0.12
Birth weight *z*-score	−0.05 (−0.73, 0.47)	−0.20 (−0.70, 0.31)	0.41 (−0.38, 0.80)	0.14
Birth length (cm)	50 (48.5, 51.5)	50 (48.12, 51.88)	50 (49, 51)	0.94
Birth length z-score	0.46 (−0.68, 0.99)	0.26 (−0.70, 1.23)	0.46 (−0.47, 0.59)	0.84
Birth head circumference (cm)	34 (33.5, 35)	34 (33.3, 35)	34 (34, 35)	0.74
Birth head circumference *z*-score	0.03 (−0.76, 0.42)	0.03 (−0.76, 0.47)	−0.11 (−0.36, 0.38)	0.81
Prostaglandin use	44/45 (98%)	33/34 (97%)	11 (100%)	>0.99
Brain volume (mL)	322.5 (298.4, 339.7)	324.0 (299.6, 337.05)	314.1 (298.4, 338.3)	0.64
Brain weight (g)	335.4 (310.3, 353.2)	336.9 (311.5, 350.5)	326.6 (310.3, 351.8)	0.64
Brain weight *z*-score	−0.59 (−1.00, −0.17)	−0.43 (−0.97, −0.16)	−0.65 (−1.15, −0.38)	0.35
Septostomy	40/45 (89%)	30/34 (88%)	10/11 (91%)	>0.99 >0.99 0.49
Pre-MRI septostomy	38/40 (95%)^a^	28/30 (93%)^a^	10/10 (100%)	
Duration septostomy to MRI	3 (2-4)	3 (2-4)	3 (2-4)	
Age at septostomy (d)	1 (1, 2)	1 (1, 2)	1 (1, 2)	0.34
Age at MRI (d)	4 (3, 5)	4 (3, 5)	4 (3, 5)	0.86
Preoperative brain injury	11 (24%)	-	11 (100%)	-
Stroke	6 (13%)	-	6 (55%)^b^	
White matter injury	5 (11%)	-	5 (45%)^b^	
Hypoxic ischemic injury	1 (2%)	-	1 (9%)	
Operative variables				
Age at surgery (d)	7 (6, 9)	7 (5.2, 8)	7 (6.5, 18.5)	0.45
Weight at surgery (kg)	3.42 (3.05, 3.64)	3.31 (3.03, 3.60)	3.60 (3.31, 3.73)	0.20
Body surface area at surgery (m^2^)	0.24 (0.21, 0.26)	0.24 (0.21, 0.26)	0.23 (0.22, 0.26)	0.65
Type of surgery				0.86
Arterial switch operation	26/45 (58%)	18/34 (53%)	8/11 (73%)	
Arterial switch operation + VSD closure	14/45 (31%)	12/34 (35%)	2/11 (18%)	
Arterial switch operation + arch reconstruction	5/45 (11%)	4/34 (12%)	1/11 (9%)	
Cardiopulmonary bypass time (min)	146 (120, 170)	152 (122, 170)	142 (121, 172)	0.68
Aortic cross-clamp time (min)	98 (83, 129)	98.5 (83, 127)	98 (76, 124)	0.85
Deep hypothermic circulatory arrest	7/45 (16%)	6/34 (18%)	1/11 (9%)	0.66
Deep hypothermic circulatory arrest time (min)	10 (3-16)	12 (7-20)	3	0.43
Selective cerebral perfusion	3/45 (7%)	2/34 (6%)	1/11 (9%)	>0.99
Selective cerebral perfusion time (min)	24 (21-27)	17, 30	24	0.71
Postoperative variables				
Intubation time (d)	3.5 (2, 6)	3 (2, 5)	4 (3, 8)	0.19
Intensive care unit time (d)	9 (6, 13.25)	8 (6, 12)	13 (9.5, 17)	0.04
Hospital time (d)	16 (12.7, 27)	15 (12, 22)	26 (16.5, 29.5)	0.07

MRI = magnetic resonance imaging; other abbreviation as in Table 1.

a Timing of septostomy was not clear based on retrospective chart review for one patient.

b 1 patient had stroke and white matter injury.

### Genetic diagnoses

Five (5/34, 15%) patients without brain injury and 3 (3/11, 27%) with brain injury had chromosome or gene abnormalities. In the group without brain injury, this included 2 patients with a deletion in chromosome 2, one patient with a chromosome 1 and CBL gene variant of uncertain significance, one patient with a TBX22 likely pathogenic variant and variant of uncertain significance in LEO1, ATP1A3, and GABRA1, and one patient with a deletion in chromosome 7 and duplication in chromosome 8. In the group with brain injury, this included one patient with a likely pathogenic variant in DSC2, one patient with a likely pathogenic variant in CHD7, and one patient with a variant of uncertain significance in the PTPN gene.

### Preoperative hemodynamics

In the subset of TGA patients with preoperative cardiac MRI, median arterial oxygen saturations were 85% at the time of MRI. Cardiac MRI findings revealed a median Qp of 7.7 L/min/m^2^, Qs of 2.4 L/min/m^2^, yielding a median ratio of pulmonary to systemic blood flow (QpQs) of 3.2 (Table 3). The highest value for pulmonary blood flow was 11.9 L/min/m^2^, while the lowest systemic blood flow was 1.1 L/min/m^2^, and the highest QpQs was 6.8.

**Table 3:** Hemodynamic Profile Stratified by Presence of Brain Injury

	Cohort (n = 45)	No Brain Injury (n = 34)	Brain Injury (n = 11)	*P* Value
Patent ductus arteriosus size (mm)	3.5 (2.5, 5)	3.5 (2.5, 5)	4 (2.5, 5)	0.946
Oxygen saturations (%)	85 (81, 90)	85 (80, 89)	85 (82, 89)	0.828
Patent ductus arteriosus flow (L/min/m^2^)	1.7 (1.1, 2.95)	1.78 (0.92, 2.94)	1.7 (1.2, 2.7)	0.895
Superior vena cava flow (L/min/m^2^)	1.31 (1.08, 1.57)	1.27 (1.08, 1.64)	1.36 (1.06, 1.42)	0.968
Ascending aortic flow (L/min/m^2^)	4.52 (3.18, 5.39)	4.64 (3.25, 5.4)	3.85 (3.15, 4.71)	0.644
Descending aortic flow (L/min/m^2^)	1.08 (0.85, 1.25)	1.06 (0.77, 1.26)	1.18 (0.93, 1.24)	0.444
Pulmonary blood flow (Qp; L/min/m^2^)	7.65 (5.88, 8.75)	7.69 (5.98, 8.62)	7.54 (5.97, 9.57)	0.682
Systemic blood flow (Qs; L/min/m^2^)	2.43 (2.01, 2.81)	2.38 (2.01, 2.86)	2.53 (2.12, 2.75)	0.771
Ratio of pulmonary to systemic blood flow (QpQs)	3.33 (2.17, 4.91)	3.36 (1.95, 4.77)	3.14 (2.32, 4.35)	0.895
Indexed cerebral blood flow (mL/min/m^2^)	542.21 (464.52, 636.99)	538.11 (465.63, 634.71)	559.46 (454.79, 635.05)	>0.99
Cerebral blood flow per 100g brain weight (mL/min/100g)	32.4 (28.22, 38.39)	31.65 (28.71, 38.03)	35.07 (29.61, 38.37)	0.756
Cerebral oxygen delivery (mL/min/m^2^)	221.72 (173.86, 266.43)	227.96 (170.34, 276.75)	219.04 (204.5, 223.66)	0.665
Ratio of superior vena cava to systemic blood flow	0.56 (0.52, 0.6)	0.56 (0.53, 0.61)	0.55 (0.51, 0.57)	0.146
Ratio of cerebral blood flow to systemic blood flow	0.23 (0.19, 0.28)	0.24 (0.18, 0.3)	0.22 (0.19, 0.25)	0.749

### TGA/IVS

**Figure 2:**
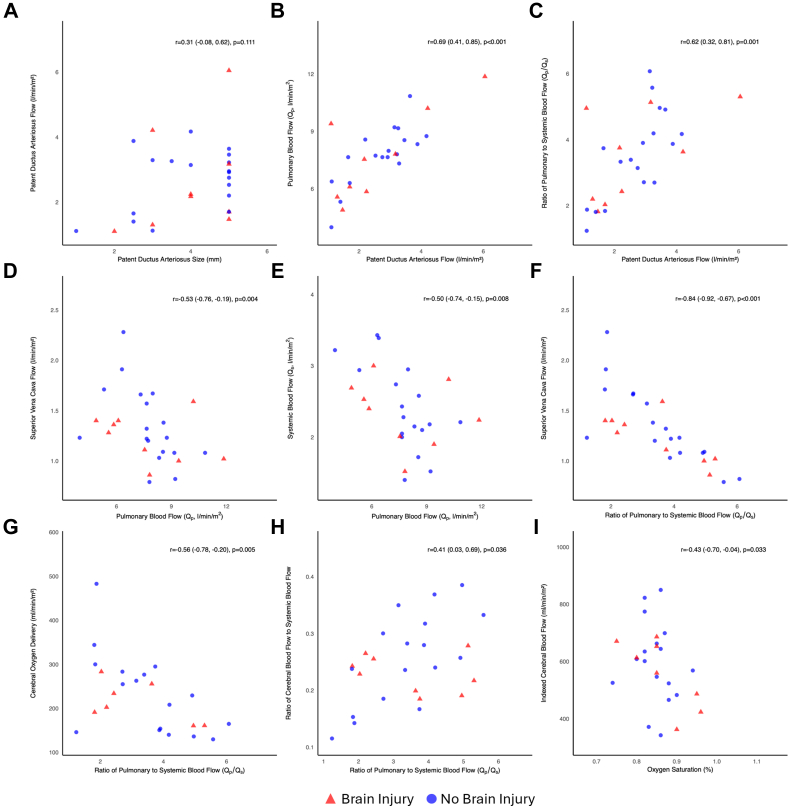
**Cardio-Cerebral Hemodynamics for Patients With TGA/IVS** (A) There was no significant association between PDA size and flow. (B) Increased PDA flow was associated with more pulmonary blood flow and (C) ratio of pulmonary to systemic blood flow. (D) Higher pulmonary blood flow was associated with a decrease in SVC flow and (E) systemic blood flow. (F) Higher ratio of pulmonary to systemic blood flow was associated with lower SVC flow, (G) lower cerebral oxygen delivery, and (H) a higher ratio of cerebral blood flow to systemic blood flow. (I) Higher oxygen saturations were associated with less cerebral blood flow. IVS = intact ventricular septum; PDA = patent ductus arteriosus; SVC = superior vena cava; TGA = transposition of the great arteries.

Correlation analysis revealed that there was no significant association between patent ductus arteriosus (PDA) size and flow (r = 0.31 [−0.08-0.62], *P* = 0.11) (Figure 2A). Increased PDA flow was associated with more Qp (r = 0.69 [0.41-0.85], *P* < 0.001) (Figure 2B) and greater QpQs (r = 0.62 [0.32-0.81], *P* = 0.001) (Figure 2C). As pulmonary blood flow increased, there was an associated decrease in SVC flow (r = −0.53 [−0.76 to −0.19], *P* = 0.004) (Figure 2D) and systemic blood flow (r = −0.50 [−0.74 to −0.15], *P* = 0.008) (Figure 2E) but no significant change in indexed cerebral blood flow (r = −0.21 [−0.55 to 0.20], *P* = 0.309). Higher QpQs was associated with lower SVC flow (r = −0.84 [−0.92 to −0.67], *P* < 0.001) (Figure 2F) and lower cerebral oxygen delivery (r = −0.56 [−0.78 to −0.20], *P* = 0.004) (Figure 2G) but not cerebral blood flow (r = −0.30 [−0.61-0.10], *P* = 0.140). Higher QpQs was also associated with a higher ratio of cerebral blood flow to systemic blood flow (r = 0.41 [0.03-0.69], *P* = 0.036) (Figure 2H). Higher oxygen saturations were associated with less cerebral blood flow (r = −0.43 [−0.70 to −0.04], *P* = 0.033) (Figure 2I) but not associated with SVC flow (r = −0.31 [−0.62-0.09], *P* = 0.125), or cerebral oxygen delivery (r = 0.08 [−0.33-0.47], *P* = 0.698).

### TGA/VSD

**Figure 3:**
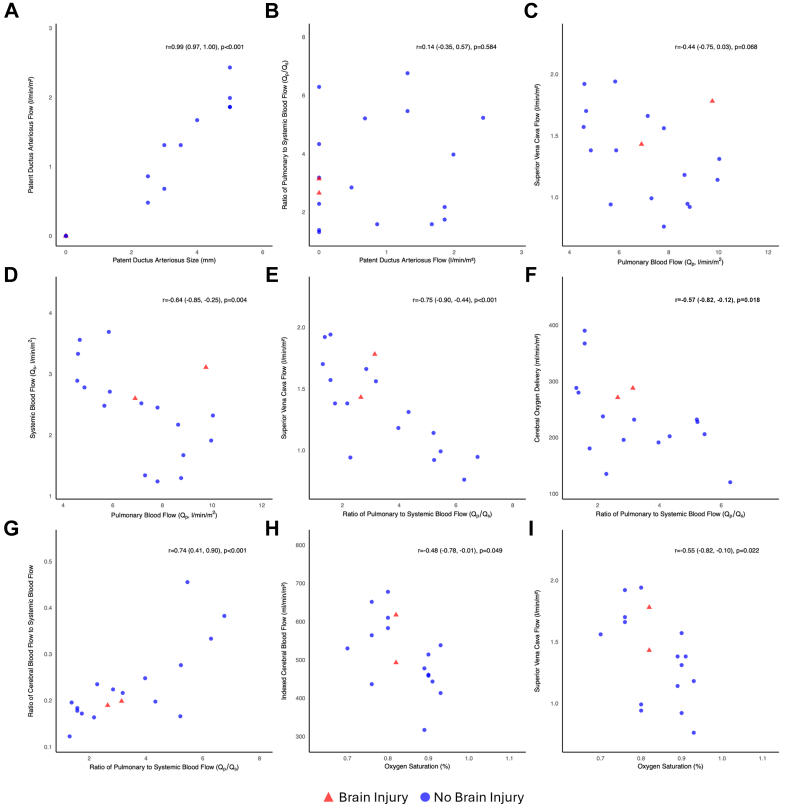
**Cardio-Cerebral Hemodynamics for Patients With TGA/VSD** (A) There was a significant association between PDA size and flow. (B) Increased PDA flow was not associated with ratio of pulmonary to systemic blood flow. (C) As pulmonary blood flow increased, there was a trend toward associated decrease in SVC flow and (D) significant decrease in systemic blood flow. (E) Higher ratio of pulmonary to systemic blood flow was associated with lower SVC flow, (F) lower cerebral oxygen delivery, and (G) a higher ratio of cerebral blood flow to systemic blood flow. (H) Higher oxygen saturations were associated with less cerebral blood flow and (I) lower SVC flow. VSD = ventricular septal defect; other abbreviations as in Figure 2.

Correlation analysis revealed that there was a significant association between PDA size and flow (r = 0.99 [0.97-1.00], *P* < 0.001) (Figure 3A). Increased PDA flow was not associated with Qp (r = 0.04 [−0.43-0.50], *P* = 0.861) or QpQs (r = 0.14 [−0.35-0.57], *P* = 0.584) (Figure 3B). As pulmonary blood flow increased, there was a trend toward associated decrease in SVC flow (r = −0.44 [−0.75-0.03], *P* = 0.068) (Figure 3C) and significant decrease in systemic blood flow (r = −0.64 [−0.85 to −0.25], *P* = 0.004) (Figure 3D) but no significant change in indexed cerebral blood flow (r = −0.25 [−0.64-0.24], *P* = 0.313). Higher QpQs was associated with lower SVC flow (r = −0.75 [−0.90 to −0.44], *P* < 0.001) (Figure 3E) and lower cerebral oxygen delivery (r = −0.57 [−0.82 to −0.12], *P* = 0.018) (Figure 3F) but not cerebral blood flow (r = −0.25 [−0.64 to 0.24], *P* = 0.315). Higher QpQs was also associated with a higher ratio of cerebral blood flow to systemic blood flow (r = 0.74 [0.41-0.90]) (*P* < 0.001) (Figure 3G). Higher oxygen saturations were associated with less cerebral blood flow (r = −0.48 [−0.78 to −0.01], *P* = 0.049) (Figure 3H) and lower SVC flow (r = −0.55 [−0.82 to −0.10], *P* = 0.022) (Figure 3I) but not cerebral oxygen delivery (r = −0.07 [−0.54 to 0.44], *P* = 0.810).

There was no difference in hemodynamic parameters between patients with and without brain injury (Table 3). While there was no clear association between hemodynamic parameters and brain injury (Figures 2 and 3), 10 of 11 (91%) patients with brain injury had QpQs > 2:1.

### Hypoxic ischemic injury

Of the 3 patients with basal ganglia or thalamic hypoxic ischemic injury, all had periods of high saturations and suspected pulmonary overcirculation (Table 4). These events occurred in the setting of unrestrictive interatrial communications and a large PDA. One of these patients was premature. Of the 3 patients with watershed hypoxic ischemic injury, all patients had a period of significant hypoxia soon after birth in the setting of restriction at the atrial septum (Table 4).

**Table 4:** Pattern of Injury and Clinical Profile of Patients With Hypoxic Ischemic Brain Injury

Patient	Diagnosis	Hypoxic Ischemic Injury Pattern	Clinical Course
Basal ganglia and thalamus			
1	Antenatal TGA/IVS	Basal ganglia and watershed	• Attempted PGE discontinuation but developed mild desaturation and PGE was restarted
			• Saturations increased to high 90s
			• PGE dose decreased but saturations continued in the high 90s
			• A few hours before arterial switch was planned, the patient presented with an episode of sudden desaturation with a decrease in peripheral perfusion and developed severe refractory shock
			• There was concern for high pulmonary blood flow and systemic steal in the setting of a large PDA
			• Patient was profoundly acidotic and needed a lot of resuscitation medication
			• DEATH: severe neurological injury
2	Antenatal TGA/IVS	Basal ganglia and thalami	• This patient did not have the same augmentation in cardiac output as others studied
			• Saturations were up to 90s (in the presence of a large PFO and PDA)
			• DEATH: decannulation following acute decompensation event post-ASO with further brain injury
3	Postnatal Preterm (28 wk) TGA/IVS	Basal ganglia and thalami	• Patient had high saturations and developed pulmonary hemorrhage. PGE dose decreased
			• Started on diuretics for tachypnea
			• PGE weaned further and discontinued with ongoing concern for pulmonary overcirculation
			• Transient periods of desaturation after decreasing/stopping PGEs
			• DEATH: withdrawal of care after diagnosis of brain injury (given prematurity and cardiac diagnosis)
Watershed			
4	Postnatal TGA/IVS	Watershed	• Significant respiratory distress and bluish discoloration with low saturations
			• Received high dose PGE, intubated, inhaled nitric oxide, and underwent BAS at 5h
			• Patient remained dusky requiring high-flow heated oxygen and had some intermittent posturing
			• Two episodes of bradycardia, apnea, and desaturation; first resolved with bagging, second after intubation
			• Extubated then reintubated for apneas and desaturation events
			• Etiology of brain injury felt to be likely multifactorial (related to hypoxia and hypoglycemia)
5	Postnatal TGA/IVS	Watershed	• Saturations in 50s on predischarge oximetry, started on PGEs
			• No acute decompensation event
			• Hypoxic ischemic injury pattern felt to be in keeping with perinatal injury
6	Antenatal TGA/IVS	Watershed	• Flat at birth
			• PGE started and intubated
			• Ongoing hypoxia so started on inhaled nitric oxide with minimal change in saturations
			• PGE increased, given epinephrine and resuscitated
			• PFO felt to be restrictive
			• Unsuccessful BAS so cannulated to extracorporeal membrane oxygenation (no loss of output or chest compressions; heart rate 50-60 beats/min during cannulation)
			• BAS performed on extracorporeal membrane oxygenation

ASO = arterial switch operation; BAS = balloon atrial septostomy; IVS = intact ventricular septum; PDA = patent ductus arteriosus; PFO = patent foramen ovale; PGE = prostaglandin E1; other abbreviation as in Table 1.

## Discussion

Significant brain injury (arterial ischemic stroke, moderate or severe white matter injury, hypoxic ischemic injury) is present in approximately 20% of newborns with TGA, approximately 3% being hypoxic ischemic injury. CMR data from our study demonstrate that preoperative patients with TGA commonly exhibit a pattern of pulmonary overcirculation accompanied by a reduction in systemic flows and cerebral oxygen delivery. Similarly, higher oxygen saturations correlated with lower cerebral blood flow, while failure of cerebral autoregulation resulting in cerebral ischemia is likely a late event in the setting of pulmonary overcirculation, and one that was not captured during these elective CMR evaluations. Although we did not demonstrate an association between this hemodynamic picture and brain injury, it is plausible that pulmonary overcirculation contributed to hypoxic ischemic injury in half of those patients exhibiting this serious pattern of brain injury. The severity of pulmonary overcirculation may be underappreciated in preoperative patients with TGA, but we propose that hypoxic ischemic injury may be preventable in this population through careful attention to oxygen saturations, sparing use of prostaglandin, and timely surgical repair (Central Illustration).

**Central Illustration:**
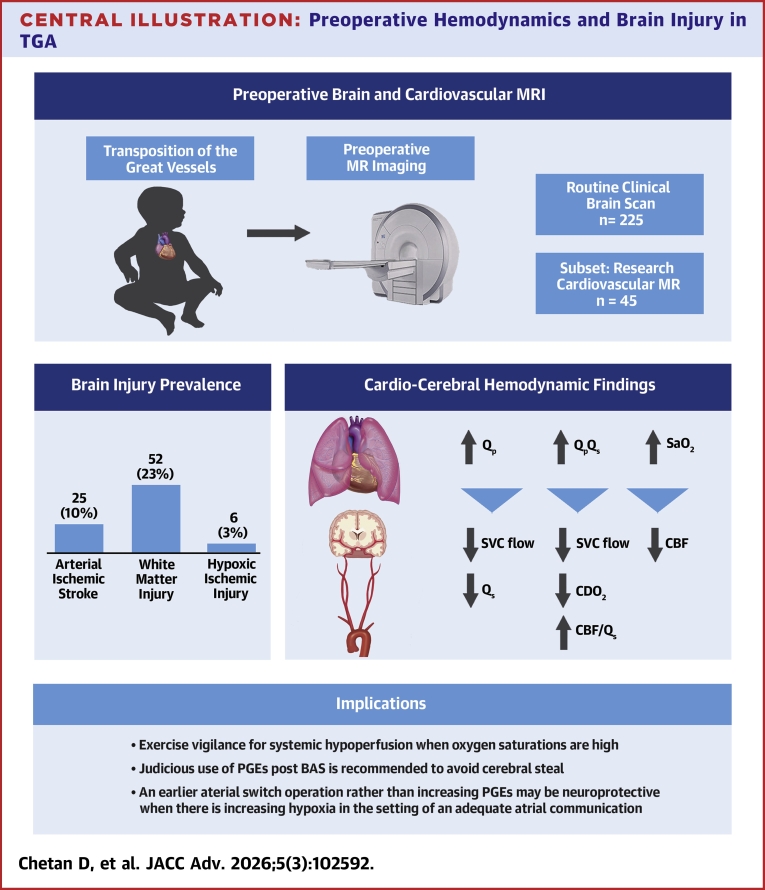
**Preoperative Hemodynamics and Brain Injury in TGA** Infants with TGA underwent routine clinical brain MRI, with a subset also receiving research cardiovascular MRI. Significant brain injury (arterial ischemic stroke, moderate or severe white matter injury, hypoxic ischemic injury) is present in approximately 20% of newborns with TGA, approximately 3% being hypoxic ischemic injury. Cardio-cerebral hemodynamics in both TGA/IVS and TGA/VSD groups showed that higher pulmonary blood flow is associated with reduced SVC and systemic blood flow. Higher ratio of pulmonary to systemic blood flow was associated with less SVC flow and cerebral oxygen delivery, with a higher ratio of cerebral blood flow to systemic blood flow. Higher oxygen saturations were associated with less cerebral blood flow. These findings highlight the importance of vigilance for systemic hypoperfusion despite apparently reassuring oxygen saturations and support cautious use of prostaglandins once an adequate atrial communication has been established. Earlier arterial switch operation can be considered to mitigate risk of brain injury. CBF = cerebral blood flow; other abbreviations as in Figure 1, Figure 2, Figure 3.

### Brain maturity and risk of brain injury in TGA

Term infants with TGA exhibit evidence of abnormal brain development with one study reporting brain maturation scores approximately 1 month younger than their gestational age.^27^^,^^28^ We have previously shown that lower late gestational fetal brain volumes were associated with an increased risk of postnatal moderate-to-severe white matter injury.^8^ Lower brain maturity scores have also been associated with an increased risk of preoperative and postoperative brain injury.^29^ In an in vivo chronic hypoxia piglet model, neuroblasts within the subventricular zone were depleted, with similar findings observed at autopsy in human congenital heart disease patients.^30^ Infants with abnormal brain microstructural and metabolic development are at increased risk for brain injury, with higher neonatal illness severity scores, lower oxygen saturations, hypotension, and septostomy all being predictive of higher brain injury scores.^31^

### Preoperative hemodynamics

The effect of a PDA on the cerebral circulation has been studied in animal models and in preterm infants. In neonatal pigs studied using 4-dimensional cine phase-contrast MRI, we observed a reduction in carotid artery flow in the presence of left-to-right shunting at the PDA.^32^ While early studies in preterm human patients similarly suggested “ductal steal,” which was thought to be involved in ischemic and hemorrhagic cerebral injury, more recent studies have shown cerebral blood flow may not be affected by PDA size. Moreover, there is no convincing evidence to suggest that PDA size is linked to brain injury in preterm infants with normal hearts.^33^, ^34^, ^35^ The impact of shunt types (atrial septal defect, VSD, PDA) on TGA circulatory physiology has been assessed using computational models, although the impact on the cerebral circulation was not assessed. The presence of a PDA was detrimental in TGA/IVS hemodynamics at baseline and in the setting of reduced pulmonary vascular resistance, with more profound hemodynamic compromise in patients with TGA/VSD with reduced pulmonary vascular resistance. In TGA/IVS, a PDA resulted in reduced systemic blood flow and systemic oxygen delivery. The effects were similar in patients with TGA/VSD but less marked at baseline because of the reduced pressure gradient between the circulations, but became more prominent when pulmonary vascular resistance dropped.^36^ In our cohort, patients with TGA/IVS with greater PDA flow had higher pulmonary blood flow and QpQs. Higher QpQs was in turn associated with lower SVC flow and cerebral oxygen delivery, suggesting a deleterious effect of greater PDA flow on patients who had already undergone atrial septostomy (90% of our cohort). With a fall in pulmonary vascular resistance, the combination of BAS and PGE results in good mixing but also high pulmonary blood flow.

Rates of BAS can differ substantially based on institutional practice variation. A report from the Pediatric Health Information System Database found rates of BAS can vary from approximately 20% to over 80%.^37^ In TGA, effective mixing between the pulmonary and systemic circulations primarily occurs at the atrial level. The left-to-right atrial shunt allows oxygenated pulmonary venous blood to enter the systemic circulation, supporting systemic oxygen delivery. The PDA can augment this mixing by increasing pulmonary blood flow and left atrial return, which enhances atrial shunting. However, while this mechanism improves systemic oxygenation, it also increases pulmonary overcirculation and can lead to a relative “steal” from the systemic circulation. Our findings confirm that as pulmonary blood flow and pulmonary-to-systemic flow ratios increase, systemic and SVC flow decrease, resulting in impaired cerebral oxygen delivery. While cerebral blood flow was preserved during our CMR examinations, we suspect that pulmonary overcirculation pushed the patients with hypoxic ischemic injury below the lower limit of cerebral autoregulation at some stage in their preoperative course. Further reductions in systemic blood flow may have been precipitated by the development of coronary steal and ventricular dysfunction.

A study using near-infrared spectroscopy and diffuse correlation spectroscopy showed lower arterial and cerebral oxygen saturations with a higher oxygen extraction fraction in patients with TGA compared to normal controls. Interestingly, no differences were found in cerebral blood volume, indexed cerebral blood flow, or cerebral oxygen metabolism index. The authors concluded that the increased oxygen extraction fraction they observed was the compensatory mechanism responsible for maintaining cerebral oxygenation.^38^ This study did not explore the differences in physiology attributable to shunts in TGA circulation.

### Hemodynamic correlation with brain injury and continuous monitoring

Correlating cerebral hemodynamics with brain injury can be challenging given temporal fluctuations in cerebral blood flow and oxygenation. Furthermore, in our study, the cardiac and brain imaging was performed at the same time such that any brain injury must have already occurred. In other words, the cardiovascular physiology recorded at the time of scan may not have reflected the hemodynamics at the time of brain injury. While our study quantified hemodynamic measures at a single time point, a proper understanding of the mechanisms of brain injury will likely require integration of longitudinal hemodynamic data that reflects the constantly evolving clinical status of the patient. An additional limitation of our approach was that neonates needed to be hemodynamically stable to undergo imaging. In future, additional noninvasive cerebrovascular monitoring modalities that provide continuous information may be useful in elucidating events that predispose to brain injury.

The NeoDoppler device was recently introduced as a monitoring technique for continuous cerebral blood flow velocity measurements in newborns.^39^ Other modalities that have been applied in this population include near-infrared spectroscopy and optical spectroscopy.^40^^,^^41^ We recently reported apparent changes in cerebral perfusion associated with white matter injury and hypoxic ischemic injury in an infant with TGA in the setting of impaired cardiac output.^42^

### Hypoxic ischemic injury

Three patients with basal ganglia/thalamic hypoxic ischemic injury all had periods of high saturation and suspected pulmonary overcirculation in the context of a large PDA. While one patient developed an episode of refractory shock, the other 2 did not have significant episodes of circulatory collapse. The presence of prematurity in one of these patients may have further predisposed to brain injury. The hemodynamic data in our study showed that significant PDA flow is related to higher oxygen saturations and lower cerebral blood flow. Basal ganglia and thalamic hypoxic ischemic injury in the term newborn is known to be due to acute hypoxia ischemia from an abrupt decrease in cerebral blood flow.^43^ This mechanism of injury wherein significant circulatory changes lead to transient but profound alterations in cerebral blood flow is consistent with our hypothesis that significant PDA flow may cause pulmonary overcirculation and a subsequent “steal” of cerebral blood flow. The mechanisms responsible for white matter injury in the setting of neonatal congenital heart disease are not entirely clear but may share a common etiology with hypoxic ischemic injury. In a preterm fetal sheep model, punctate periventricular lesions characterized by microcystic necrosis affecting selectively vulnerable populations of preoligodendrocytes that resemble those seen in newborns with congenital heart disease have been induced by a combination of maternal hypoxia and intermittent ischemia produced with temporary carotid artery occlusion.^44^

### Important implications

Key takeaways for the preoperative management of newborns with TGA include:

1. In patients waiting for arterial switch operation after BAS, judicious use of PGEs is recommended to avoid the risk of “cerebral steal.”
2. Exercise vigilance for signs of systemic hypoperfusion in preoperative TGA with high oxygen saturations.
3. For the infant that has an adequate atrial communication and is becoming more hypoxic, earlier arterial switch rather than increasing PGE may be a neuroprotective option.

Further studies integrating continuous hemodynamic and cerebral blood flow monitoring will be helpful in corroborating these findings and further elucidating the causes of brain injury. More investigation into less conventional approaches such as the Ukrainian experience with early arterial switch operation during the first few hours of life may also help to reveal targets for neuroprotection as this approach minimizes the duration of abnormal circulatory physiology.^45^

### Study limitations

In this single-center study, the rate of BAS was high, which likely affected preoperative hemodynamics, potentially resulting in our population being at greater risk of pulmonary overcirculation. The relatively low sample size of patients undergoing CMR resulted in only one of the hypoxic ischemic injury patients having CMR data. Further research employing a multicenter study design and continuous cerebral monitoring and neurodevelopmental follow-up might provide additional insights into the mechanisms of brain injury in preoperative patients with TGA.

## Conclusions

In the modern era, brain injury remains common in preoperative patients with TGA. Although no clear associations were found between preoperative hemodynamics and brain injury, this study provides evidence that higher QpQs was associated with lower SVC flow and cerebral oxygen delivery and may predispose infants with TGA to hypoxic ischemic injury and white matter injury. Our findings suggest that to preserve cerebral blood flow, preoperative TGA patients who remain hypoxemic after BAS may be better served by earlier arterial switch operation rather than attempting to increase PDA size with PGEs. They also suggest that in addition to hypoxia secondary to poor mixing, a second mechanism involving pulmonary overcirculation in the setting of PDA following BAS may also result in significant hypoxic ischemic brain injury in newborns with TGA. While rare, our data suggest this important complication may be avoidable with judicious use of PGE, careful hemodynamic monitoring, and routine repair in the first few days after birth (Central Illustration).

## Funding support and author disclosures

This work was supported by the 10.13039/501100000024Canadian Institutes of Health Research (MOP-142204). The authors have reported that they have no relationships relevant to the contents of this paper to disclose.
