# Supplementary material for: Preoperative Hemodynamics and Brain Injury in Transposition of the Great Arteries
Source: JACC Adv. 2026 Mar 25;5(3):102592. doi: 10.1016/j.jacadv.2026.102592 (PMC13351995; doi:10.1016/j.jacadv.2026.102592)
Supplement: Supplemental_Material [file mmc1.docx]

**SUPPLEMENTAL TABLE 1. DETAILED DIAGNOSES**

|  | Cohort  (n=285) | No CMR  (n=240) | CMR  (n=45) | p |
| --- | --- | --- | --- | --- |

| TGA IVS  + Coarctation  + Small Muscular VSD | 152 (53%)  1 (0%)  4 (1%) | 125 (52%)  1 (0%)  4 (2%) | 26 (58%)  1 (2%)  - | 0.66 |
| --- | --- | --- | --- | --- |
| TGA VSD  + Arch Hypoplasia +/- Coarctation  + LVOTO  + PS and Borderline RV  + Interrupted Aortic Arch (B) | 76 (27%)  11 (4%)  11 (4%)  1 (0%)  1 (0%) | 62 (26%)  8 (3%)  11 (5%)  1 (0%)  1 (0%) | 14 (31%)  3 (7%)  -  -  - |  |
| DORV TGA  + PA/PS  + AP Window  + Double Aortic Arch  DORV ncVSD straddling MV | 1 (0%)  5 (2%)  1 (0%)  1 (0%)  1 (0%) | 1 (0%)  5 (2%)  1 (0%)  1 (0%)  1 (0%) | -  -  -  -  - |  |
| Taussig Bing  + Arch Hypoplasia  + Coarctation + Transverse Arch Hypoplasia  + Arch Hypoplasia + CoA + hypoplastic RV  + Interrupted Aortic Arch (A)  + Interrupted Aortic Arch (B) | 4 (1%)  1 (0%)  9 (3%)  1 (0%)  1 (0%)  1 (0%) | 4 (2%)  -  9 (4%)  1 (0%)  1 (0%)  1 (0%) | -  1 (2%)  -  -  -  - |  |
| cAVSD (Primum) + VA discordance  uAVSD + TGA + Arch Hypoplasia | 1 (0%)  1 (0%) | 1 (0%)  1 (0%) | -  - |  |

**SUPPLEMENTAL TABLE 2. SURGICAL DETAILS**

|  | Cohort  (n=285) | No CMR  (n=240) | CMR  (n=45) | p |
| --- | --- | --- | --- | --- |
| Arterial Switch Operation  + VSD Closure  + PA band (subsequent VSD closure)  + Arch Reconstruction  - Previous Arch Reconstruction  + Aortic Arch Reconstruction  + PA band (subsequent VSD closure)  + Subsequent BCPC (1.5V Repair)  + PA band (subsequent VSD closure)  + AVSD repair | 153 (54%)  77 (27%)  2 (1%)  20 (7%)  1 (0%)  2 (1%)  4 (1%)  1 (0%)  2 (1%)  1 (0%) | 127 (53%)  63 (26%)  2 (1%)  16 (7%)  1 (0%)  1 (0%)  4 (2%)  1 (0%)  2 (1%)  1 (0%) | 26 (58%)  14 (31%)  -  4 (9%)  -  1 (2%)  -  -  -  - | 0.92 |
| Yasui  Rastelli  + 1.5V Repair  - Previous Vegetation Excision and BCPC  (subsequent 1.5V repair with Rastelli)  Nikaidoh | 1 (0%)  9 (3%)  2 (1%)  1 (0%)  4 (1%) | 1 (0%)  9 (4%)  2 (1%)  1 (0%)  4 (2%) | -  -  -  -  - |  |
| AP Window Repair, RVPA conduit | 1 (0%) | 1 (0%) | - |  |
| Died before Surgery | 4 (1%) | 4 (2%) | - |  |

**SUPPLEMENTAL TABLE 3. BRAIN MR PARAMETERS**

| **Sequence** | **Type** | **TE, ms** | **TR, ms** | **FOV, mm** | **Matrix size, mm** | **Slice thickness, mm** | **B-value** |
| --- | --- | --- | --- | --- | --- | --- | --- |
| **T1WI** | 3D | 3 | 1920 | 200 x 200 x 200 | 256 x 256 | 0.8 | - |
| **Axial T2WI** | 2D | 210 | 9970 | 140 x 140 x 114 | 218 x 320 | 4 | - |
| **Axial DWI** | 2D | 97 | 11000 | 213 x 213 x 213 | 192 x 192 | 4 | 800 |
| **Axial SWI** | 2D | 40 | 50 | 200 x 200 x 119 | 213 x 448 | 2 | - |
| **MR Venography*** | 3D | 13.3 | 75 | 220 x 151 x 123 | 99 x 192 | 1.1 | - |

*Phase Contrast Velocity 7cm/s
